# High-Quality Development of Science and Technology Finance and Logistics Industry in the Yangtze River Economic Belt: Coupling Analysis Based on Deep Learning Model

**DOI:** 10.1155/2022/5185190

**Published:** 2022-08-21

**Authors:** Lingrui Li, Xianjun Huang, Pengfei Liu

**Affiliations:** School of Economics and Management, Anqing Normal University, Anqing, Anhui 246133, China

## Abstract

Using the entropy method, the coupling coordination model, and the Tobit model, the coupling coordination degree of the high-quality development of science and technology finance and the logistics industry in the Yangtze River Economic Belt in China from 2009 to 2020 is measured, and its influencing factors are found. The study found that the overall coupling coordination degree of the Yangtze River Economic Belt has shown a rapid upward trend; the development gap of the interprovincial coupling coordination degree has a narrowing trend. Except for Sichuan Province, the average coupling coordination degree decreases from the downstream to the upstream; the mechanism analysis shows that the coupling effect of the two. There are scale effect, innovation effect, talent effect, and structure effect; the analysis of influencing factors shows that innovation effect and talent effect have the most obvious promoting effect on the coupled and coordinated development of the two. In addition, the upgrading of the industrial structure, the effective driving of science and technology, the improvement of the logistics foundation, and the further development of finance also have a positive effect on it. Finally, according to the conclusions, suggestions are put forward from five aspects: insisting on innovation, talent training, risk prevention, policy orientation, and industrial structure upgrading, so as to realize the further coupling of high-quality development of technology finance and logistics industry.

## 1. Related Introduction

Science and technology finance refers to a series of financial policies, tools, and services that the government, enterprises, financial markets, and social financial institutions provide capital elements for scientific and technological innovation activities and can promote the coordinated development of related industries. As China's economy enters a new stage of development, as an organic combination of technological innovation and finance, science and technology finance guides capital, digital, and other elements to gather in the “sunrise industry” and then enables the high-quality development of China's economy. It is generally recognized by the world. As the “lubricant” of China's modern commercial and trade circulation system and the “accelerator” of economic growth, the logistics industry is also the inherent requirement and service basis for the construction of a “dual circulation” economic pattern. Some studies have found that the development of the financial industry is good for modern logistics. Industry development has a codirectional linkage effect. Industrial integration and innovation development is a realistic path to realize my country's economic structural reform in the new era, economic and other aspects of coordinated and full development. However, scholars mostly consider the possible impact of technology finance on regional innovation capabilities, corporate innovation performance, industrial structure upgrading, and regional economy from a single perspective, the coupling and coordination between the high-quality development of the logistics industry and technological finance has not yet been paid more attention by scholars.

“Coupling” refers to “the interaction between two or more complex subsystems,” and “coordination” refers to “the benign interaction between subsystems.” Therefore, if we want to clarify the coupling and coordination relationship between the high-quality development of science and technology finance and the logistics industry, we must first clarify the interaction between the two subsystems of science and technology finance and the logistics industry and then explore the benign influence relationship between the subsystems. Whether from the perspective of industrial coordinated development or from the perspective of microenterprise operation, previous studies have not thoroughly considered the possible impact of new development concepts such as innovation-driven and green development on the coupling effect. In fact, in the process of implementing coordinated development, there are still some problems in the coupling effect under the impact of unfavorable factors such as the impact of the epidemic and the frequent occurrence of international disputes. Does the degree of coupling effect match the needs of my country's economic development? What is the path through which this coupling effect is achieved? What are the factors that affect the coupling effect? In view of my country's leading exploration position in the field of science and technology finance and the era requirements of high-quality economic development in the new era, the discussion of the above issues will help clarify the coupling and coordination role of science and technology finance and the high-quality development of the logistics industry, which has certain practical significance and theoretical value.

The marginal contributions of this paper mainly include the following two aspects: first, a comprehensive evaluation index system for the high-quality development of science and technology finance and logistics in the Yangtze River Economic Belt is constructed, and the entropy method and coupling coordination theory are used to measure the coupling coordination degree. The comprehensive evaluation index can more deeply describe the comprehensive development differences between the two subsystems: the high-quality development of science and technology finance and the logistics industry. Second, this study attempts to analyze the coupling mechanism of the high-quality development of science and technology finance and the logistics industry and finds that there are scale effects, innovation effects, talent effects, and structural effects in the coupling effect of science and technology finance and the high-quality development of the logistics industry; factor analysis, using the Tobit model, found that the innovation effect and the talent effect have a significant role in promoting the coordinated development of the two. The industrial structure upgrade, the strengthening of the transportation network construction, the further development of finance, and the effective transformation of scientific and technological achievements into competitiveness are the same. It has a positive role in promoting the coupling and coordinated development of the two. The conclusions of this research will help relevant decision makers understand the current coupling trend of high-quality development of technology finance and logistics industry and provide a certain reference for future policy positioning and related domestic research [1–10].

## 2. Related Work

According to the theory of new economic geography, along with the improvement of the transportation network foundation, that is, the development of the logistics industry, the degree of trade liberalization of goods and services can be effectively improved, and the further development of other industries can be driven. At the same time, the effective cross-regional flow of labor is also based on the high-quality development of the logistics industry. The agglomeration of high-quality human capital will also drive industrial and regional economic development. According to the theory of economic growth, under the background that my country's economy has entered a new stage of development, the economic growth model has changed from the extensive drive of capital, labor scale expansion, and technological progress to the innovation drive dominated by technological and organizational innovation. Schumpeter's innovation theory also points out that organizational innovation and technological innovation are the two sources of enterprise development and economic growth, while the extension of organizational innovation to the macroeconomic field is reflected in the reconstruction of industry, capital, and even infrastructure. Based on the above theories, the high-quality development of the logistics industry, as the “arterial pipeline” of economic development, and the science and technology finance, as a powerful “engine” of technological innovation and economic development, should have a certain coupling effect in the current economic development situation. Following this research idea, this paper attempts to introduce the theory of system coupling. Based on the two subjects participating in the coupling effect, it analyzes the coupling mechanism of the high-quality development of science and technology finance and the logistics industry from two aspects and further explores the high-quality development of science and technology finance and the logistics industry.

**Figure 1:**
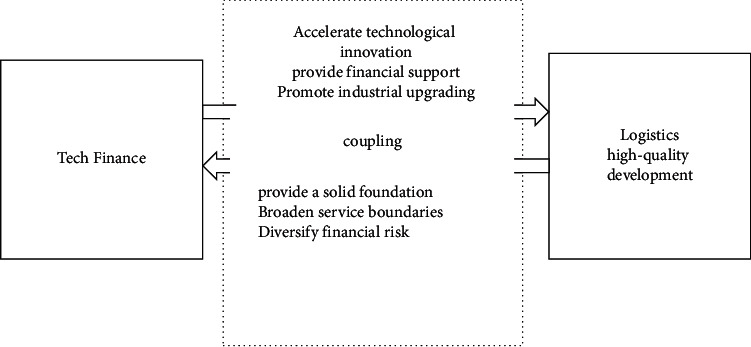
Coupling mechanism of technological finance and high-quality development of logistics industry.

Based on this, this paper constructs a diagram of the coupling mechanism of technological finance and the high-quality development of the logistics industry, as shown in Figure 1.

Based on the above analysis, there is a scale effect in the coupling and coordination of high-quality development of science and technology finance and the logistics industry. Technology finance promotes the concentration of capital elements in the high-tech fields of the logistics industry, such as cold chain logistics, which can promote the large-scale growth of scientific and technological achievements in the logistics industry and promote the high-quality development of the logistics industry. At the same time, the intensive and intelligent high-quality development of the logistics industry also provides a solid real economic foundation for the development of science and technology finance. The benign coupling of the two can play a benign role of 1 + 1 > 2 in China's economic growth. The second is the innovation effect. Technological finance and logistics industry need innovation drive to promote high-quality development. This is not only an inevitable requirement for high-quality development but also the driving force behind the high-quality development of technology finance and logistics. The third is the talent effect. First, technology finance can provide comprehensive service talents for the development of the logistics industry. Second, the high-quality development of the logistics industry will promote the orderly flow of human capital across regions. This will further promote the high-quality development of the technology finance and logistics industries. The last is the structural effect. The adjustment and upgrading of the industrial structure of the two is conducive to the rational allocation of capital and other elements, thereby promoting the coordinated development of the two [10–15].

## 3. Deep Learning Model Methods

### 3.1. Entropy Value Method

#### 3.1.1. De-Dimensionalization

Since the dimensions of the above indicators are inconsistent, in order to eliminate the influence caused by the difference between the dimension and the order of magnitude, the selected indicators are de-dimensioned. In order to ensure the validity of the calculation data, Min(*W*_*i*，*j*_) take 0.99 times and Max(*W*_*i*，*j*_) 1.01 times. The specific processing process is as follows:

(1) $$\begin{array}{c} \text{Positive indicators: }W_{i,j}^{\prime }=\frac{W_{i,j}-0.99min\left(W_{i,j}\right)}{1.01max\left(W_{i,j}\right)-0.99min\left(W_{i,j}\right)}, \end{array}$$

(2) $$\begin{array}{c} \text{Inverse indicators: }W_{i,j}^{\prime }=\frac{1.01max\left(W_{i,j}\right)-W_{i,j}}{1.01max\left(W_{i,j}\right)-0.99max\left(W_{i,j}\right)}. \end{array}$$

In equations (1) and (2), *i* represents the *i*th year (*i* = 1, 2, 3,…, *n*), and *j* represents the *j*th observation (*j* = 1, 2, 3,…, *m*). *W*_*i*,*j*_′ is the *W*_*i*,*j*_ normalized value, Min(*W*_*i*,*j*_) is *W*_*i*,*j*_ the minimum value, and Max(*W*_*i*,*j*_) is *W*_*i*,*j*_ the maximum value.

#### 3.1.2. Calculation of Indicator Weights

The first step is to calculate the indicator contribution value *X*_*i*,*j*_:

(3) $$\begin{array}{c} X_{i,j}=\frac{W_{i,j}^{'}}{\sum_{i=1}^{n}W_{i,j}^{'}}. \end{array}$$

The second step is to calculate the information entropy *E*_*j*_:

(4) $$\begin{array}{c} E_{j}=\text{k}\sum_{i=1}^{n}X_{i,j}\ln\;X_{i,j}, \end{array}$$

where *k*=−1/ln  *n*.

The third step is to calculate the redundancy *D*_*j*_:

(5) $$\begin{array}{c} D_{j}=1-E_{j}. \end{array}$$

The fourth step is to calculate the indicator weight *Y*_*j*_:

(6) $$\begin{array}{c} Y_{j}=\frac{D_{j}}{\sum_{j=1}^{m}D_{j}}. \end{array}$$

#### 3.1.3. Calculation of Comprehensive Evaluation Index

Using the linear weighting method to calculate the comprehensive evaluation index of high-quality development of science and technology finance and logistics industry *Z*_*j*_:

(7) $$\begin{array}{c} Z_{i,j}=\sum_{j=1}^{m}Y_{j}W_{i,j}^{\prime }. \end{array}$$

In formula (7), the comprehensive evaluation index of science and technology finance is recorded as *Z*_*A*_, and the comprehensive evaluation index of high-quality development of the logistics industry is recorded as *Z*_*B*_.

### 3.2. Coupling Degree, Coordination Index, and Measurement of Coupling Coordination Degree

In the coupling and coordination degree model between two systems, the coupling degree measures the interaction between the two subsystems, and the comprehensive coordination index measures the degree of harmony between the two subsystems. This study builds a coupling degree and a comprehensive coordination index model to measure the degree of coupling and coordination between technology finance and the high-quality development of the logistics industry.

#### 3.2.1. Coupling Degree C of Technology Finance and High-Quality Development of Logistics Industry

The coupling model is constructed as follows:

(8) $$\begin{array}{c} \text{C}=2\times \frac{\sqrt{Z_{A}Z_{B}}}{Z_{A}+Z_{B}}. \end{array}$$

In formula (8) , *C* is the coupling degree, and C ∈ [0，1]. When *C* is close to 1, it indicates that the interaction between the technology financial system and the high-quality development system of the logistics industry is stronger. However, the coupling degree can only be used to measure the interaction strength between two subsystems and cannot reflect the coordination degree of complex systems. In order to avoid *Z*_*A*_ the *Z*_*B*_ situation where both are low, but *C* is high. This study constructs a comprehensive coordination index model to reflect the overall coordination degree between the financial subsystem of science and technology and the subsystem of high-quality development of the logistics industry.

#### 3.2.2. Comprehensive Coordination Index F of High-Quality Development of Technology Finance and Logistics Industry

The comprehensive coordination index model is constructed as follows:

(9) $$\begin{array}{c} \text{F}=\alpha Z_{A}+\beta Z_{B}. \end{array}$$

In formula (9), *F* is the comprehensive coordination index, *α*, *β* is the undetermined coefficient, which represents the importance and contribution value of the subsystem to the overall complex system. In this study, it is believed that the financial technology subsystem and the logistics industry high-quality development subsystem have the same status, so it is generally believed *α*=*β*=0.5.

#### 3.2.3. Coupling and Coordination Degree of Technology Finance and High-Quality Development of Logistics Industry

The coupling coordination degree model is constructed as follows:

(10) $$\begin{array}{c} \text{T}=\sqrt{C\times F}. \end{array}$$

In Formula (10), *T* is the coupling coordination degree between the financial technology subsystem and the logistics high-quality development subsystem, and *T* ∈ [0, 1]. *C* is the coupling degree of the two subsystems, and F is the comprehensive coordination index of the two subsystems. According to common practice, this study is divided into three stages according to the size of *T* value. The specific criteria are shown in Table 1.

## 4. Coupling Analysis Based on Deep Learning Model

### 4.1. Data Sources and Processing

The data of this study come from “China Transportation Statistical Yearbook,” “China Financial Statistical Yearbook,” “China Science and Technology Statistical Yearbook,” “China Tertiary Industry Statistical Yearbook,” “China Energy Statistical Yearbook,” “China Statistical Yearbook,” National The Bureau of Statistics, the seventh census data, the CEADS database, and the statistical yearbooks of provinces and cities use the imputation method for missing values [16].

### 4.2. Research Objects

This study selects the Yangtze River Economic Belt region as the research object. As of the end of 2020, its area is about 2.0523 million square kilometers, accounting for 21.4% of the national area; the permanent population is 603 million, accounting for 42.9% of the national population; the total GDP in 2020 is 47.16 trillion yuan, accounting for 46.4% of the value of the country's total GDP for that year. On the one hand, its comprehensive advantages lie in its current economic and political status and its huge future development potential. Second, it has unique geographical advantages. The region where water transportation is developed, the road network is vertical and horizontal, and the resource endowment such as economy, population, and technology ranks first in the country as a whole. The logistics industry has a solid infrastructure in this region, and technology finance also has a solid real economic foundation here. Finally, the high level of science and education in the region has a massive talent pool, which can support the local economic development. But on the other hand, a review of its internal findings shows that although the regional comprehensive strength is strong, the provinces and cities have shown differentiated development. Not only the human factors, geographical and natural environment factors are different but also the status quo and structure of industrial development are different. Therefore, there may also be regional differences in the degree of coupling between technological finance and the high-quality development of the logistics industry. Therefore, taking the Yangtze River Economic Belt as the research object, measuring the coupling level of high-quality development of science and technology finance and logistics industry has a certain regional representativeness.

### 4.3. Construction of Indicator System

#### 4.3.1. Comprehensive Evaluation System for the Development of Science and Technology Finance

When building a comprehensive evaluation system for the development of science and technology finance, some studies start from the endowment of science and technology finance and design an index system from the dimensions of science and technology finance resources, financing, funding, and output (Cao Hao et al., 2011). Drawing on previous research practices, this study comprehensively considers the principles of data availability, reliability, and measurability and builds a comprehensive evaluation index system for science and technology finance around the four dimensions of financial scale, technological foundation, financial environment, and R&D innovation. The financial scale is measured by the added value of the financial industry, the market value of the stock market, and the premium income of insurance institutions. The scientific and technological foundation is measured by the number of Internet broadband accesses, the length of optical cable lines, and financial and technological expenditures. The financial environment is measured by four indicators, including the ratio of banking financial institutions' loans to local GDP. The level of R&D innovation is measured by three indicators, including the number of regional invention patents. The specific indicators are defined as shown in Table 2 [17].

#### 4.3.2. Evaluation System for High-Quality Development of Logistics Industry

In order to reflect the high-quality development of the logistics industry, the technological innovation level of the logistics industry is measured by four indicators, including the number of traded contracts in the regional technology market and the full-time equivalent of research and development personnel. Fourth, in the implementation of the new development concept, the high-quality development of the logistics industry should be a multidimensional balanced development. At present, problems such as unbalanced, insufficient, and uncoordinated development still exist in the development process of the logistics industry, especially in terms of green development. Therefore, the energy structure of the logistics industry is measured by the CO_2_ emissions and terminal energy consumption of the logistics industry, and the green transportation structure of the logistics industry is measured by the railway operating mileage. When designing specific evaluation indicators, this study defines the transportation, warehousing, and postal industries as the logistics industry, as shown in Table 3 [18].

## 5. Analysis of Measurement Results

### 5.1. Time Series Trend of Coupling Coordination Degree in the Yangtze River Economic Belt

**Figure 2:**
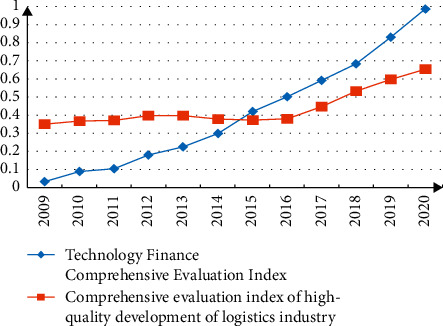
Time trend of coupling coordination degree of the Yangtze River Economic Belt from 2009 to 2020.

According to formulas (1)–(10), the data of 11 provinces and cities in the Yangtze River Economic Belt are substituted for calculation, and the comprehensive evaluation index and coupling of the high-quality development of science and technology finance and logistics industry in the Yangtze River Economic Belt from 2009 to 2020 are obtained. The degree of coordination, the degree of comprehensive coordination, and the degree of coupling coordination are shown in Figure 2 and Table 4, respectively [19].

From the perspective of the development level of subsystems, the overall development level of science and technology finance in the Yangtze River Economic Belt and the comprehensive development level of the logistics industry have been greatly improved. In particular, the comprehensive development level of technology finance has rapidly developed from 0.03 in 2009 to 0.98 in 2020. Since 2012, the development level of technology finance has accelerated significantly. This may be due to the proliferation of digital technology, which has given strong momentum to the development of financial technology. The comprehensive development level of the logistics industry has also increased significantly since 2016. This may be due to the official release of my country's “Outline of the Development Plan for the Yangtze River Economic Belt” in 2016, and the policy orientation has promoted the rapid development of the logistics industry. In addition, from 2009 to 2014, the development level of the logistics industry has always been higher than the development level of science and technology finance, which belongs to the advanced development type of logistics. From 2015 to 2020, the development level of science and technology finance is higher than the comprehensive development level of the logistics industry, which belongs to the advanced financial development type.

From the perspective of the overall development trend from 2009 to 2020, the coupling degree, coordination degree, and coupling coordination degree of the high-quality development of science and technology finance and logistics industry in China's Yangtze River Economic Belt have shown a rapid upward trend, but the coupling degree is greater than the coordination degree. In 2009, the coupling coordination degree was only 0.3332, which was in the stage of mild detuned recession. As of 2020, the coupling coordination degree has risen to 0.8967, an increase of 1.72 times. During this period, the high-quality coupled development of science and technology finance and the logistics industry evolved from a stage of unbalanced decline to a stage of coupled and coordinated development. It has successively crossed the four types of dysregulation recession, barely coordinated development, primary coordinated development, and intermediate coordinated development from a mild imbalanced recession type to a well-coordinated development type, which is only one step away from the ultimate goal of high-quality coordinated development. In the future, we should strengthen the coupling relationship between science and technology finance and the high-quality development of the logistics industry, implement the goal of science and technology finance serving the high-quality development of the logistics industry, deepen the feedback effect of the high-quality development of the logistics industry on science and technology finance, and realize the high-quality coupled development of the two [20, 21].

### 5.2. Spatial Distribution Differences of Coupling Coordination Degree in the Yangtze River Economic Belt

According to formulas (1)–(10), the data of 11 provinces and cities in the Yangtze River Economic Belt are substituted for calculation, and the coupling coordination of high-quality development of science and technology finance and logistics industry in 11 provinces (cities) in the Yangtze River Economic Belt from 2009 to 2020 is obtained, as shown in Table 5.

There are considerable interprovincial and regional differences in the coupling effect of high-quality development of science and technology finance and logistics industry in the Yangtze River Economic Belt, but the interregional differences tend to narrow. It can be seen from Table 5 that the coupling coordination degree interval of the 11 provinces and cities in the Yangtze River Economic Belt has been [0.2758, 0.8549] since 2009, and the interval in 2020 is [0.3516, 0.7766], which shows that the regional development gap is further narrowing. Judging from the average ranking, the three provinces and cities of Jiangsu, Shanghai, and Zhejiang in the lower reaches of the Yangtze River Economic Belt have the highest level of development of coupling coordination. It is followed by Sichuan, Anhui, Hubei, Hunan, and Jiangxi in the middle reaches, and Chongqing, Yunnan, and Guizhou in the lowest order. From 2009 to 2020, the coupling coordination degree of Zhejiang and Jiangsu provinces has increased slightly, while the coupling coordination degree of Shanghai has regressed from the good coupling coordinated development type to the primary coupling coordinated development type but still remains in the coupled coordinated development stage. This may be due to the fact that Shanghai's financial industry was repeatedly impacted by unfavorable factors such as international trade frictions and financial market turmoil between 2009 and 2020. Anhui Province and Hubei Province showed a slight growth trend, evolving from a type of near-disordered decline to a type of reluctantly coordinated development; Sichuan Province has always maintained a type of reluctantly coordinated development; Jiangxi and Guizhou have shown a steady growth trend, both of which have leaped one category upward. The coupling coordination degree of Chongqing and Yunnan basically remains unchanged and is in the stage of imbalance and weakening. The development of the coupling coordination degree of the 11 provinces and cities in the Yangtze River Economic Belt has shown a steady and progressive development trend as a whole.

In 2009 and 2020, the coupling and coordinated development of the upstream and downstream of the Yangtze River Economic Belt is significantly different in terms of the current development level and growth rate. According to the upper, middle, and lower reaches of the Yangtze River Economic Belt, the coupling coordination degree of the Yangtze River Economic Belt basically shows a decreasing trend in the order from the lower reaches to the upper reaches. Except for Sichuan Province, the level of coupling and coordination between the high-quality development of science and technology finance and the logistics industry decreases from east to west. In the Yangtze River Economic Belt, in 2009, the development level of the coupling coordination degree of Guizhou Province and Jiangxi Province was lower than that of the surrounding provinces and cities. However, in 2020, the coupling coordination level gap between the upstream and downstream provinces and cities in the Yangtze River Economic Belt has narrowed. In Guizhou Province and Jiangxi Province, development is more obvious.

As shown in Table 6, there are considerable differences in the development level of science and technology finance and the development level of the logistics industry in 11 provinces and cities, and the interprovincial gap in the development level of science and technology finance is slowly expanding, while the interprovincial gap in the high-quality development level of the logistics industry gradually shrink. This study selects the cross-sectional data of four time nodes in 2009, 2012, 2015, 2018, and 2020 for comparative analysis. The study found that, on the one hand, the high-quality development of the logistics industry needs to be focused on. From 2009 to 2020, the comprehensive development level of Shanghai's logistics industry ranked first among 11 provinces and cities. This is due to Shanghai's advantageous geographical location and unique political and economic advantages in the opening-up economy, so it is ahead of other provinces and cities in terms of logistics infrastructure. However, in the current economic situation, with the increasing international trade friction and the requirements of China's domestic circular economy, Shanghai is facing the challenge of the dual transformation of high-quality development of technology finance and logistics. Thanks to the leading breakthroughs in e-commerce, digital finance, and other fields, Zhejiang Province has maintained a good growth rate in both technological finance and high-quality development of the logistics industry. As of 2020, its comprehensive development level of technology finance ranks first among 11 provinces and cities, and its high-quality development level of the logistics industry ranks second. On the other hand, although the growth rate of science and technology finance in the middle and upper reaches is higher than that of the high-quality development of the logistics industry, due to the low development level of the financial industry in the provinces and cities in the middle and upper reaches in 2009, the overall development level of science and technology finance is lower than that of the logistics industry. High-quality development level: the deep integration and development of technology finance and the logistics industry in the middle and upper reaches of the country needs to complement the shortcomings, strengthen the infrastructure construction of science and technology finance, and narrow the gap between the development of science and technology finance in the upstream regions. At the same time, we must learn from the development experience of Jiangsu and Shanghai provinces and cities, pay attention to digital risks and financial risks, and implement the coordinated development of technology finance and logistics industry.

## 6. Conclusion

The study found that, first, from the overall trend, the coupling and coordination of the high-quality development of science and technology finance and the logistics industry in the Yangtze River Economic Belt has shown a rapid growth trend. From 2009 to 2011, its coupling coordination was in a mild dysregulation recession—the type on the verge of dysregulation recession, from 2011 to 2015, it was on the verge of dysregulation recession—the primary coordinated development type, and from 2015 to 2018, it was in the primary coordinated development—the intermediate coordinated development type. From 2018 to 2020, it is in the middle-level coordinated development—the type of good coordinated development. In the future, we should further deepen the coupling effect of high-quality development of science and technology finance and logistics industry and strive to make the coupling effect of high-quality development of science and technology finance and logistics industry in my country's Yangtze River Economic Belt enter the type of high-quality coordinated development as soon as possible. Second, from the perspective of interprovincial regional differences, there are obvious differences in the degree of coupling and coordination among 11 provinces and cities, but the development gap in the degree of interprovincial coupling and coordination is gradually narrowing. Divided according to the upper, middle and lower regions, except for Sichuan, the average coupling coordination degree decreases from east to west, and from downstream to upstream. The three provinces and cities of Guizhou, Yunnan, and Chongqing are limited by their own endowments, and the logistics industry and technology finance have poor foundations and slow development. In the downstream area, Shanghai needs to find opportunities for transformation and breakthroughs in my country's current “dual circulation” pattern and make full use of its own solid infrastructure advantages to achieve high-quality development of technology finance and logistics. Third, from the perspective of the differences in the development of subsystems, the comprehensive level of high-quality development of the logistics industry showed a steady growth trend during the sample period, and the gap between the development levels among provinces was gradually narrowing. However, its growth rate is not as fast as that of science and technology finance. It is necessary to further strengthen the transformation of scientific and technological achievements, attach importance to the introduction of talents, and implement innovation-driven implementation. Benefiting from the diffusion and application of information technology, technology finance has grown rapidly during the sample period and has evolved from an extremely imbalanced recession type to a high-quality coordinated development type. After 2015, the development level of technology finance exceeds the high-quality development level of logistics. However, it is worth noting that the gap between the development of science and technology finance in the middle and upstream regions and the development of science and technology finance in the downstream regions is further widening. At the same time, the financial risk supervision issues hidden behind the rapid growth of upstream regions also need further attention. When the external financial environment is turbulent, it should ensure its own stable development. Fourth, the mechanism analysis combined with the analysis of influencing factors shows that there are scale effect, innovation effect, talent effect, and structure effect in the coupling effect of the two; strengthening the role of innovation effect and talent effect in the coupling relationship can significantly promote the coordinated development of the two. In addition, the upgrading of the industrial structure, the improvement of the logistics foundation, the effective transformation of scientific and technological achievements, and the further development of finance all have a positive role in promoting the coupled and coordinated development of the two.

## Figures and Tables

**Table 1:** Definition of coupling coordination stages and types.

Coupling coordination stage	Coupling coordination degree *T* interval	Coupling coordination type
Dissonance decline development 0 ≤ T< 0.4_	[ 0.00, 0.10)_	extreme dissonance recession
	[0.10, 0.20)	Severe dissonance recession
	[0.20, 0.30)	Moderate dissonance decline
	[0.30, 0.40)	Mild dysregulation decline

Over-conciliation development 0.4 ≤ T< 0.6_	[0.40, 0.50)	On the verge of a dysfunctional recession
	[0.50, 0.60)	Barely coordinated development

Coupling and coordinated development 0.6 ≤ T≤ 1.0	[0.60, 0.70)	Primary coordinated development
	[0.70, 0.80)	Intermediate coordinated development
	[0.80, 0.90)	Well-coordinated development
	[0.90, 1.00]	High-quality coordinated development

**Table 2:** Comprehensive evaluation index system for the development of science and technology finance.

Dimension	Evaluation indicators	Unit	Direction
Financial scale	Regional financial industry value added	Billion	Just
	Regional insurance agency premium income	Billion	Just
	Regional stock market capitalization	Billion	Just

Science and technology foundation	The number of internet broadband access in the region	Ten thousand	Just
	Regional fiber optic cable length	Kilometer	Just
	Regional financial technology expenditure	Billion	Just

Financial environment	Year-end financial loan balance of regional banking industry/GDP	%	Just
	Regional financial industry urban employed personnel/urban employed personnel	%	Just
	Number of legal entities in the regional financial industry	Individual	Just
	Regional digital financial inclusion index^1^	—	Just

Innovating	Regional invention patents authorized	Item	Just
	Regional technology market contract turnover/financial industry added value	%	Just
	Number of regional R&D institutions	Individual	Just

**Table 3:** Comprehensive evaluation index system for high-quality development of logistics industry.

Dimension	Evaluation indicators	Unit	Direction
Service output	The added value of the regional logistics industry	Billion	Just
	Regional cargo turnover	100 million tons/km	Just
	Regional courier volume	10,000 pieces	Just
	Regional logistics industry added value/tertiary industry added value	%	Just

Factor input	Regional logistics industry fixed asset investment/tertiary industry fixed asset investment	%	Just
	The number of legal entities in the regional logistics industry/the number of legal entities in the tertiary industry	%	Just
	Employed persons in urban units of regional logistics industry/employed persons in urban units	%	Just

Technological innovation	The number of contracts in the regional technology market	Item	Just
	Regional research and experimental development staff full-time equivalent	People/year	Just
	Number of regional logistics technology institutions	Individual	Just
	The amount of foreign technology imported by the region	Item	Just

ECO development	_2_emissions from the regional logistics industry	Million tons	Burden
	Regional final energy consumption of logistics industry	Tons of standard coal	Burden
	Regional business mileage (railway)	10,000 kilometers	Just

**Table 4:** The coupling and coordination degree of high-quality development of science and technology finance and logistics industry in the Yangtze River Economic Belt in 2009–2020.

Years	Coupling C	Comprehensive coordination degree F	Coupling coordination degree T	Coupling coordination type
2009	0.5748	0.1932	0.3332	Mild dysregulation decline
2010	0.7911	0.2287	0.4253	On the verge of a dysfunctional recession
2011	0.8274	0.238 0	0.4437	On the verge of a dysfunctional recession
2012	0.9263	0.2908	0.519 0	Barely coordinated development
2013	0.9606	0.3122	0.5476	Barely coordinated development
2014	0.9929	0.3396	0.5806	Barely coordinated development
2015	0.9985	0.3977	0.6302	Primary coordinated development
2016	0.9906	0.4409	0.6608	Primary coordinated development
2017	0.9906	0.5203	0.7179	Intermediate coordinated development
2018	0.9926	0.6068	0.7761	Intermediate coordinated development
2019	0.9867	0.7143	0.8395	Well-coordinated development
2020	0.9798	0.8207	0.8967	Well-coordinated development

**Table 5:** Coupling and coordination degree of high-quality development of science and technology finance and logistics industry in 11 provinces (cities) from 2009 to 2020.

Provinces and cities	2009	2010	2011	2012	2013	2014	2015	2016	2017	2018	2019	2020	Mean rank
Jiangsu	0.7414	0.8063	0.8176	0.8225	0.8165	0.7949	0.7852	0.7874	0.7925	0.7398	0.7582	0.7649	1
Shanghai	0.8549	0.8115	0.7778	0.7613	0.7358	0.7077	0.7003	0.7235	0.7158	0.7036	0.689 0	0.6805	2
Zhejiang	0.7 001	0.7497	0.7294	0.7093	0.7198	0.7129	0.7244	0.734 0	0.7281	0.745	0.7638	0.7766	3
Sichuan	0.5382	0.5319	0.5315	0.5313	0.5327	0.5583	0.5465	0.5445	0.5301	0.5506	0.5384	0.5501	4
Anhui	0.4985	0.5063	0.5126	0.5153	0.5254	0.5295	0.5367	0.5744	0.5781	0.5344	0.5429	0.543 0	5
Hubei	0.4777	0.4777	0.4667	0.4819	0.5125	0.5289	0.5511	0.5548	0.5426	0.5063	0.5058	0.509 0	6
Hunan	0.4674	0.4573	0.468 0	0.4546	0.4541	0.4543	0.4444	0.4325	0.4473	0.4764	0.494	0.4795	7
Jiangxi	0.3911	0.3866	0.3808	0.3694	0.3835	0.3969	0.3932	0.4051	0.4374	0.4604	0.4819	0.4521	8
Chongqing	0.4238	0.4296	0.4044	0.3931	0.4152	0.4338	0.4068	0.4111	0.3834	0.3708	0.3947	0.4298	9
Yunnan	0.3636	0.3365	0.3292	0.3346	0.3484	0.359 0	0.3536	0.356 0	0.3494	0.3174	0.3433	0.3647	1 0
Guizhou	0.2758	0.2532	0.2728	0.2569	0.2904	0.2968	0.2971	0.303 0	0.325 0	0.3229	0.3515	0.3516	1 1

**Table 6:** Comprehensive evaluation index of high-quality development of technology finance and logistics industry in 2009, 2012, 2015, and 2020.

Provinces and cities	2009		2012		2015		2018		2020	
	*Z *_A_	*Z *_B_	*Z *_A_	*Z *_B_	*Z *_A_	*Z *_B_	*Z *_A_	*Z *_B_	*Z *_A_	*Z *_B_
Anhui	0.213	0.289	0.225	0.313	0.241	0.343	0.249	0.327	0.281	0.309
Jiangsu	0.667	0.453	0.830	0.551	0.748	0.507	0.726	0.412	0.762	0.449
Shanghai	0.737	0.724	0.541	0.62 0	0.442	0.543	0.492	0.497	0.441	0.485
Zhejiang	0.625	0.383	0.583	0.434	0.625	0.44 0	0.718	0.428	0.770	0.472
Hubei	0.221	0.235	0.194	0.278	0.279	0.329	0.251	0.261	0.256	0.262
Hunan	0.167	0.284	0.138	0.308	0.157	0.248	0.168	0.306	0.207	0.254
Jiangxi	0.095	0.244	0.072	0.256	0.092	0.259	0.117	0.383	0.151	0.276
Guizhou	0.044	0.130	0.036	0.118	0.046	0.167	0.081	0.133	0.107	0.142
Sichuan	0.299	0.280	0.240	0.331	0.263	0.338	0.305	0.301	0.309	0.296
Yunnan	0.110	0.157	0.085	0.147	0.075	0.206	0.060	0.168	0.084	0.208
Chongqing	0.150	0.214	0.118	0.201	0.121	0.225	0.112	0.167	0.160	0.212

## Data Availability

The dataset can be accessed through the corresponding author upon request.
